# Ground Thermal Diffusivity Calculation by Direct Soil Temperature Measurement. Application to very Low Enthalpy Geothermal Energy Systems

**DOI:** 10.3390/s16030306

**Published:** 2016-02-29

**Authors:** José Manuel Andújar Márquez, Miguel Ángel Martínez Bohórquez, Sergio Gómez Melgar

**Affiliations:** Escuela Técnica Superior de Ingeniería, Universidad de Huelva, Ctra. Palos de la Ftra.-Huelva s/n, Huelva 21819, Spain; andujar@uhu.es (J.M.A.M.); sergio.gomez@dimme.uhu.es (S.G.M.)

**Keywords:** very low enthalpy geothermal energy, soil temperature, ground thermal diffusivity, ground temperature probe, instrumentation system

## Abstract

This paper presents a methodology and instrumentation system for the indirect measurement of the thermal diffusivity of a soil at a given depth from measuring its temperature at that depth. The development has been carried out considering its application to the design and sizing of very low enthalpy geothermal energy (VLEGE) systems, but it can has many other applications, for example in construction, agriculture or biology. The methodology is simple and inexpensive because it can take advantage of the prescriptive geotechnical drilling prior to the construction of a house or building, to take at the same time temperature measurements that will allow get the actual temperature and ground thermal diffusivity to the depth of interest. The methodology and developed system have been tested and used in the design of a VLEGE facility for a chalet with basement at the outskirts of Huelva (a city in the southwest of Spain). Experimental results validate the proposed approach.

## 1. Introduction

Geothermal exchange technology takes advantage of the thermal energy stored in the surface area of the Earth (first 100 m). Up to 10–15 m deep approximately, ground heat is supplied by the sun and rain. From there the underground temperature increases about 3 °C per 100 m depth, due to the internal thermal energy of the Earth. On average, the underground temperature at 10 m depth remains constant throughout the year and substantially equal to the average temperature of the place [1,2,3,4].

**Figure 1:**
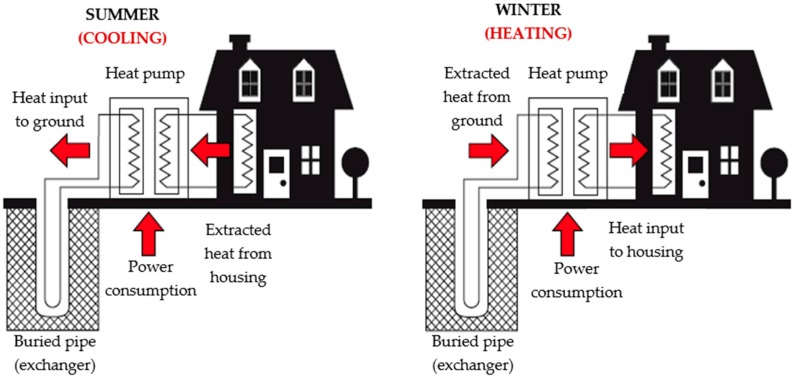
Typical operation of a geothermal exchange system with heat pump.

Initially geothermal exchange systems were developed for heating in cold climates, hence its development in northern European countries, USA and Canada, but they are also suitable for cooling, increasing their profitability and interest in countries of southern latitudes. In cooling mode, heat is extracted from the building and this is transferred to the ground. In heating mode, heat is extracted from the Earth and it is transferred to the building (Figure 1). Of course the underground temperature remains unchanged (or it does so very slightly depending on depth), but the room temperature is what changes.

Geothermal energy is called very low enthalpy (very low enthalpy geothermal energy, VLEGE) when the heat transfer from the ground occurs at low depth and low temperature (usually less than 40 °C) [1]. To exploit effectively the heat capacity of the ground, a heat-exchanger system has to be constructed [5]. In practice, heat exchange is performed by internal fluid flowing through a collector (buried pipe), which is usually water, glycol water or even air. The geothermal exchange can be performed through a heat pump [6], whereupon the internal and external circuits of the building become independent (see Figure 1), or directly by circulating the geothermal fluid through the network building’s HVAC (heating, ventilating, and air conditioning) by floor heating, radiators or air outlets for example.

As it is well known the coefficient of performance (COP) of a heat pump is inversely proportional to the temperature difference of the heat sources [2,4,7]; in the case of the VLEGE it is not big and substantially independent of seasonal variations. Even so the COP of the heat pump of a VLEGE system is around 4, which means that the system captures an average of 3 kWh of thermal energy from the ground per kWh consumed from the mains supply to power the heat pump. For this reason VLEGE is a very competitive alternative compared with conventional alternatives for heating and cooling. Furthermore from the environmental point of view the impact of a VLEGE system is much lower than a conventional air conditioning system.

**Figure 2:**
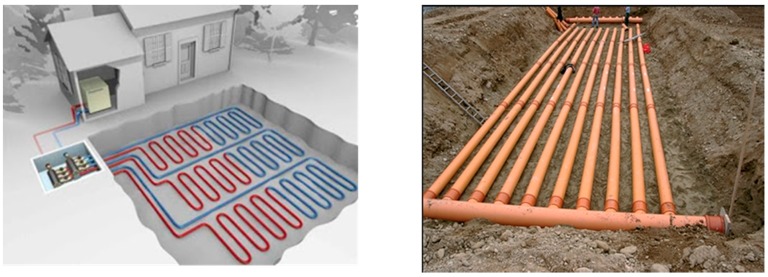
Collectors typical arrangement in a VLEGE facility.

Since VLEGE uses the heat accumulated in the more superficial layers of the Earth, collectors are usually placed horizontally adopting different shapes (Figure 2) and trying always to have the largest surface area in contact with the ground [8]. The sizing of these collectors is carried out according to the thermal performance of the soil, which depends on its composition, density, water content and of course on the depth. The two key soil parameters for designing a VLEGE system are the *soil temperature* at the collectors depth *T*(*z*) and the *ground thermal diffusivity* at that depth $\left(\alpha_{z}\right)$. By definition *thermal diffusivity*, *α* (m^2^/s), is the thermal conductivity divided by density and specific heat capacity at constant pressure, and it represents the ability of a material to conduct thermal energy relative to its ability to store thermal energy. Since ground thermal diffusivity depends on soil type, density and water content, the range of values that can take is very broad. For instance in [9] ground thermal diffusivity values ranging from $1.72\times 10^{-6}\;m^{2}/\text{s to }3\times 10^{-6}\;m^{2}/s$, depending on the soil characteristics. Another example: for a sandy soil with a density of $1.46\times 10^{3}\;\text{Kg}/m^{3}$, [10] provides values ranging between $0.3\times 10^{-6}\;m^{2}/\text{s to }1.1\times 10^{-6}\;m^{2}/s$, depending on its water content. If we continue researching on the specialized literature, we can find very different value ranges. This is because even in the same soils (same composition), if their density and/or moisture degrees are different their α values as well. This means that for a given soil and depth, α can varies throughout the year, depending on rainfall. Therefore it makes sense to work with average values.

Assuming that the soil behavior is uniform (for stricter mathematical study beyond the scope of this paper can be found [11] among other references), which as we know is not true in practice, ground thermal diffusivity values are tabulated according to the soil composition, so ground thermal diffusivity can be estimated by tables. In a more realistic way, ground thermal diffusivity can be measured in the laboratory from a test tube of the ground or performing a thermal response test (TRT) [12,13,14].

A TRT is an *in-situ* measurement method which is currently the most exact way to determine the ground effective thermal properties; it is the most reliable and the only recommended for large or medium VLEGE facilities, however it is a complex and expensive method. Therefore, the TRT is impractical to carry out in most VLEGE facilities (which are small and domestic) so that designers resort to using tables. This implies an approach to the composition, water content and compaction of the ground at the depth where the collectors are buried; consequently the probability of making mistakes can be high. Incorrect design of the VLEGE facility (diameter and collector length, type of material, type of fluid and heat exchanger) cause malfunction and poor performance of the heat pump. This is a serious problem: dig up collectors and change them is expensive and sometimes, depending on the characteristics of the facility, impossible. Consequently, this may result in the need to use external heating/cooling, which call into serious question the energy efficiency of the facility.

This paper develops a methodology and instrumentation system for the indirect measurement of the thermal diffusivity of a ground at a given depth from measuring its temperature at that depth. The methodology and developed system have been tested and used in the design of a VLEGE facility for a chalet with basement at the outskirts of Huelva (a city in the southwest of Spain).

The paper is laid out as follows: Section 2 explains the theoretical foundation that develops the methodology for calculating the ground thermal diffusivity at a given depth from knowledge of the ground temperature at that depth. Section 3 explains the developed hardware/software instrumentation system for measuring ground temperature at a given depth accurately and at low cost. Section 4 is devoted to experimentation in a real scenario and obtained results; these demonstrate the usefulness of the proposed solution. Finally, Section 5 provides some conclusions about the work done and its results.

## 2. Theoretical Foundation

The energy from solar radiation and other atmospheric agents is continuously transferred to the surface of the Earth, which causes effects on its temperature in depths close to the surface. The observed soil temperature at various depths has been studied decades ago [15,16,17,18]. Since then, no new theoretical approaches in this field are found in literature, but obviously what has evolved with technological advancement are measuring systems and data processing.

It is well known that the typical annual cycles of monthly average soil temperatures at the surface and at depths close to the surface, follow a pattern of easy fit to a simple harmonic function. Then, the temperature in the soil surface can be represented by the following function:

(1) $$T_{soil\;surface}\left(t\right)=T_{m}-T_{p}\cos\left(\omega t-\varphi \right)$$

where:

$T_{m}=$ Annual average temperature of soil in the stable layer; it is commonly set to the average temperature of air, practically the average temperature in the place (°C).

$T_{p}=$ Amplitude (°C); the peak deviation of the function from zero. In this case the annual amplitude of the monthly average temperature cycle in the place.

t= Time coordinate (s). To set a starting time (*t* = 0), it begins to run from 1 January at 0 s. This way of measuring the time obviously result in a phase shift since the beginning of the sinusoid in general will not coincide with *t* = 0.

ω= Angular frequency (rad/s). The rate of change of the function argument in units of radians per second; ω=2π/T, where *T* is the period of the sinusoid; in this case the annual temperature cycle, *i.e.*, $T=365.242189\times 24\times 3600=3.1557\times 10^{7}$ s.

φ= Phase (rad). When φ is non-zero, the entire waveform appears to be shifted in time by the amount φ/ω seconds. A negative value represents a delay, and a positive value represents an advance.

The soil temperature oscillation near the surface for the stand point of heat conduction theory has been discussed since decades in various texts of heat transfer [19,20,21]. The subsurface is treated as a semi-infinite domain, $Ω=\left\{x={\left(x,y,z\right)}^{T}:-\infty <x,y<\infty ,0\leq z<\infty \right\}$. The subsurface temperature, T(x,t), at any point x and time *t* is provided by the heat conduction equation:

(2) $$\rho C\frac{\partial T\left(x,t\right)}{\partial t}=\nabla \cdot \left[k\nabla T\left(x,t\right)\right]$$

where ρ is the average soil density (Kg/m^3^), C the soil specific heat capacity (J/Kg·K) and k the soil thermal conductivity (W/m·K). The three soil parameters ρ, C and k, vary, to different degrees, in space and time due to soil heterogeneity and changing water content. Soil anisotropy gives rise to the thermal conductivity tensor k, whose principle components are aligned with the coordinate system, such that the off-diagonal components of this tensor are *K_ij_* = 0 for i≠j. Equation (2) is subject to an initial condition:

(3) $$T\left(x,0\right)=T_{m}$$

This temperature varies in response to atmospheric fluctuations at the ground surface (z=0), which manifest themselves through the boundary condition (1): T(x,y,0,t). By solving the heat equation in the transient state for a semi-infinite medium whose surface temperature is imposed by Equation (1), the soil temperature *versus* depth only can be obtained:

(4) $$T_{soil}\left(z,t\right)=T_{m}-T_{p}e^{-z\sqrt{\frac{\omega }{2\alpha }}}\cos\left(\omega t-\varphi -z\sqrt{\frac{\omega }{2\alpha }}\right)$$

where z is the depth (m) and α is the ground thermal diffusivity (m^2^/s) given by:

(5) $$\alpha =\frac{k}{\rho C}$$

Equation (4) allows us to show some important features of the evolution of soil temperature with depth:

- As the depth increases the second term of Equation (4) tends to zero, which means that the soil temperature tends to the annual average in the place ($T_{m}$).
- The amplitude of the temperature variation decreases exponentially with depth.
- The phase shift increases with depth.

**Figure 3:**
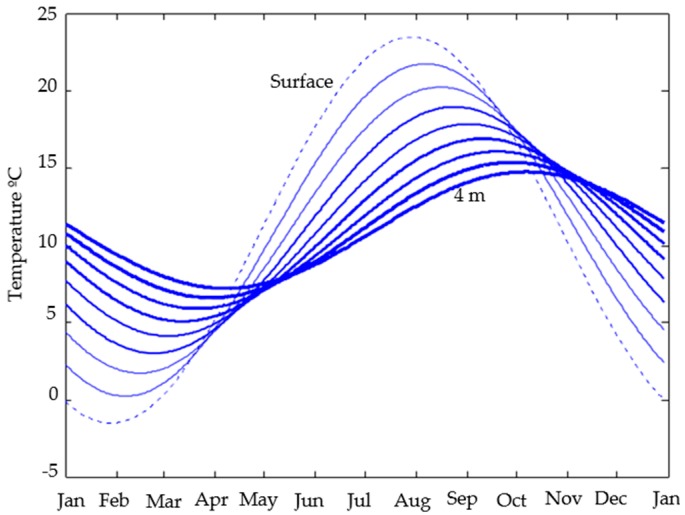
Typical temperature behavior of the soil close to the surface at different depths. The curves show the evolution of the temperature from the surface (0 m) to 4 m depth in steps of 0.5 m.

As an illustrative example, let’s consider a place where the annual average temperature is 11 °C ($T_{m}$ in Equation (4)), the annual peak to peak amplitude of the monthly average temperature cycle $\left(T_{pp}\right)$ is 25 °C $\left(\text{then }T_{p}\text{ in Equation }\left(4\right)\text{ is }25/2=12.5{}^{\circ}C\right)$, and whose physical soil characteristics, constant regardless of the depth, are: *ρ* = 2000 Kg/m^3^, *k* = 2 W/(m·K) and *C* = 99 J/(Kg·K). The application of Equation (4) to this place allows obtaining the temperature behavior of the soil at different depths. This is shown in Figure 3, where the behavior of the soil temperature is drawn from the surface to a depth of 4 m in steps of 0.5 m.

Note in Figure 3 that, as the depth increases, the amplitude of thermal fluctuations decreases, and their maximum and minimum are going phased out (due to the thermal inertia of the soil itself). At 4 m deep, the phase shift reached two months. As the depth increases annual fluctuations in soil temperature are diminishing and it is getting closer to the annual average of the place. In the real world, and for a practical case, in depths between the surface and about 10 m, the temperature of the soil varies depending on the depth and anything that disturbs the type, compactness and uniformity of the ground and of course moisture or groundwater (anything that changes the ground thermal diffusivity). This means that in general the ground thermal diffusivity will not be constant, whereby the distribution of the actual curves may not be as uniform as in Figure 3. This actual behavior goes unnoticed when considering the ground thermal diffusivity of a soil at a given depth from tables, since it is considered uniform.

From Equation (4) and considering a ground thermal diffusivity obtained by tables for example, we can calculate the evolution of the soil temperature at any depth between the surface and the deep where the soil temperature tends to the annual average in the place $\left(T_{m}\right)$, *i.e.*, around 10 m. Nevertheless we know that ground thermal diffusivity is not constant, which prevents knowing the degree of accuracy in the calculation of the temperature at the considered depth. But what can we do if we have precise ground temperature measurements at that depth?

From Equation (4), the minimum ($T_{L}$) and maximum ($T_{H}$) temperatures of the soil that take place during the annual cycle for any depth (z) can be calculated. This is:

(6) $$T_{L}=T_{m}-T_{p}e^{-z\sqrt{\frac{\omega }{2\alpha }}}$$

(7) $$T_{H}=T_{m}+T_{p}e^{-z\sqrt{\frac{\omega }{2\alpha }}}$$

From any of the above two equations the ground thermal diffusivity at any depth *z* can be calculated. For example from Equation (7):

(8) $$\alpha_{z}=\frac{\omega }{2}{\left(\frac{z}{\text{ln }\frac{T_{p}}{T_{H}-T_{m}}}\right)}^{2}$$

$T_{m}$ and $T_{p}$ values are easy to know from meteorological stations or by measuring at place, so if you can measure accurately $T_{H}$ over annual cycle for a z depth, Equation (8) allows obtaining the ground thermal diffusivity without need to make estimates by tables or calculating it by the costly and complex TRT. Similar reasoning could be made with respect to $T_{L}$. Certainly $\alpha_{z}$ can vary along a seasonal cycle (to find instantaneous values you can use Equation (4)), but in the absence of extreme situations such as flooding for example, at depths far of the surface (the interest for VLEGE) there is less influence of surface weather, therefore under normal conditions the ground thermal diffusivity will have an average value close to that obtained by Equation (8).

## 3. Developed Instrumentation System for Measuring Ground Temperature

**Figure 4:**
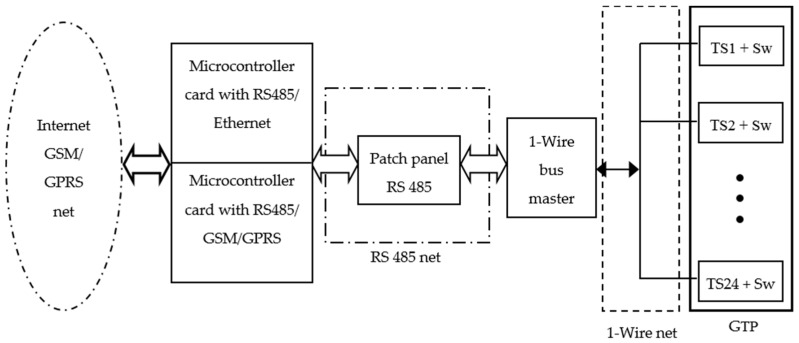
Block diagram of the developed instrumentation system for measuring ground temperature.

Figure 4 shows the block diagram of the developed instrumentation system for measuring ground temperature. The system is monitored and controlled remotely by two microcontroller cards via a virtual instrument (VI) from anywhere with an Internet connection. Now we will be explaining each of the blocks.

### 3.1. Ground Temperature Probe

**Figure 5:**
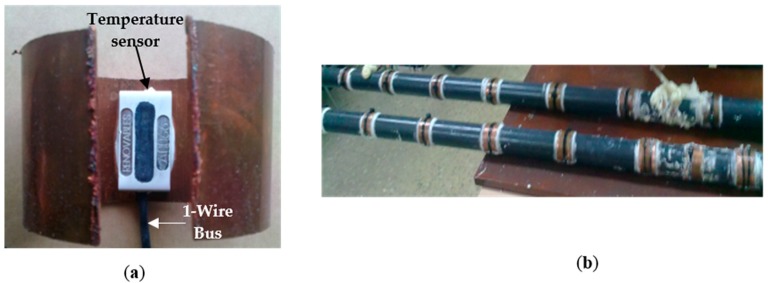
(**a**) Temperature sensor attached to the metal ring; (**b**) GTP disassembled into two sections and arrangement of the sensors on the probe.

For continuous measurement of the temperature of the ground at different depths, a ground temperature probe (GTP) was designed and built using a PVC pipe of 5 m length and 100 mm diameter. In it, 24 temperature sensors (TS1–TS24) with digital output and a 1-wire bus connection (patented by the authors, [22]) are placed. Each TS can be connected/disconnected to the 1-wire bus by an addressable electronic switch (Sw) which function will be explain later in this section. TS are spaced 20 cm each, and they are glued to a metal ring to favor the thermal transmittance between ground and sensor (see Figure 5a). Metal rings are glued to the pipe and securely fastened by a polyethylene clamp (see Figure 5b). All wiring flows inside the pipe and it is filled with polyurethane foam to TS protect. 24 TS are located from level 0.2 m to 4.80 m deep. The first 20 cm of GTP must be above the surface to facilitate connections and recovery, and at the tip of the GTP it is not advisable to place TS as it is the part that suffers most in the introduction to the field. The GTP can be disassembled into two sections of 2.5 m for easy transport (see Figure 5b). Of course we could have another GTP length; however it has done so for constructive interest of the house where the experiment was conducted. That is, when a chalet or house with basement is built, an excavation of approximately 3 m around the perimeter of the house is required. From this depth it is easier and cheaper to dig about 2 m further by a backhoe loader. Therefore the maximum depth to build at low cost a VLEGE facility in a chalet or house with basement is around 5 m. Of course you can build the VLEGE facility deeper; however this requires a work infrastructure which makes much more expensive the construction. In any case, obviating the construction work details that are not the subject of this paper, developed system experimentation (see Section 4) was carried out with ground temperature measurements between 0 and 4.80 m depth.

The 24 TS are connected via 1-wire bus (see Figure 4 and Figure 5a). This is a low-cost bus for digital communication over twisted-pair cable with 1-wire components. As each TS has a unique 64-bit serial code, it is possible to connect multiple TS to the same 1-wire bus quickly, simply and very cheaply. Using the code from each TS, its temperature measurement can be addressed from a PC or microcontroller board. The 1-wire bus is not a bus designed to operate in industrial environments (where noise can be an important factor to consider) because it is not differential. In [23] it is shown as providing robustness to the 1-wire bus and how to connect the TS to the bus.

**Figure 6:**
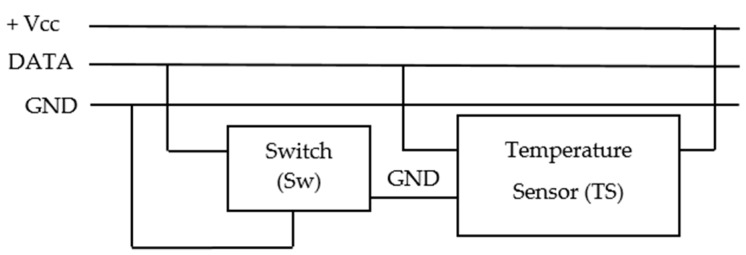
1-Wire bus connection of the temperature sensor + switch set.

Regarding the connection of sensors in the GTP and its electronic control, a robust solution it was implemented as required for our system. With respect to TS communications via the 1-wire bus, if one of the connected TS fails, the rest of the bus can become unusable. When this occurs in a facility of easy access, the damaged TS is changed and the problem is solved. However, for our application is a major problem because to solve it would have to dig up the GTP. In order to prevent and resolve this potential problem, each TS can be connected/disconnected to the 1-wire bus by an addressable electronic switch (Sw). In Figure 6 detail of TS + Sw connection of Figure 4 is shown. The switch has been implemented by the DS2413 integrated circuit and allows physically disconnecting the TS of the 1-wire bus by the GND line.

The scheme of Figure 6, also serves another important function: when a TS branch is connected to a 1-wire bus there is a mismatch impedance at the branch point which can cause reflections to the bus. To avoid it, a 100 Ω series resistor between DATA and GND lines on each TS should be placed. The internal resistance of the switch fulfills this task, avoiding the need to place an additional external resistance [24].

### 3.2. The 1-Wire Bus Master

A detailed explanation of the 1-wire bus master (see Figure 4) can be found in [23]; it is designed around PIC 18F252 and is able to addressing simultaneously two 1-wire networks of 50 TS each. For this application we are only using 24 sensors, so that the same 1-wire bus master also applies for deeper studies where more sensors would be required.

### 3.3. The RS 485 Patch Panel

The RS 485 patch panel (see Figure 4) has the function to allow connection to multiple sensors (room temperature, irradiance, speed and direction wind, humidity, *etc.*) or other devices to the system. In this way a single communications channel as that of Figure 4 can monitor multiple variables, although the measurement points are spaced hundreds of meters.

Choosing the RS 485 bus is based on its robust features in terms of noise performance (it is a differential bus) and its ability to carry data over long distances.

### 3.4. Microcontroller Cards

The two microcontroller cards (see Figure 4) are based on Arduino, an open-source prototyping platform based on easy-to-use hardware and software. These cards control communications with devices connected to patch-panel and store measured data in a SD memory. In addition they manage online external communications either via Ethernet or GSM/GPRS. Although data are sent in real time, they are also stored in one of the microcontroller cards for safety, so if for any reason communications fail, always be possible recover the data. The system is designed so that in those locations that do not have Internet connection, you can use the mobile communications GSM/GPRS. In the worst case, if not even there is mobile phone coverage we can always use a radio modem link. This last case is not included in Figure 4.

### 3.5. Monitoring and Control Software

For full monitoring and control of the developed instrumentation system, we have developed a VI in LabVIEW^TM^, which is located on a computer at the headquarters of the research group to which the authors belong, at the Higher Technical School of Engineering of the University of Huelva. In this way the whole facility operates as a remote lab [25].

The VI consists of different sub VI with screens that display real time information. It also enables data processing for several kinds of operations and also provides multiple graphs types. The data can be processed by the VI itself or exported in various formats like spreadsheet for example. This will be discussed in more detail in Section 4 of this manuscript.

**Figure 7:**
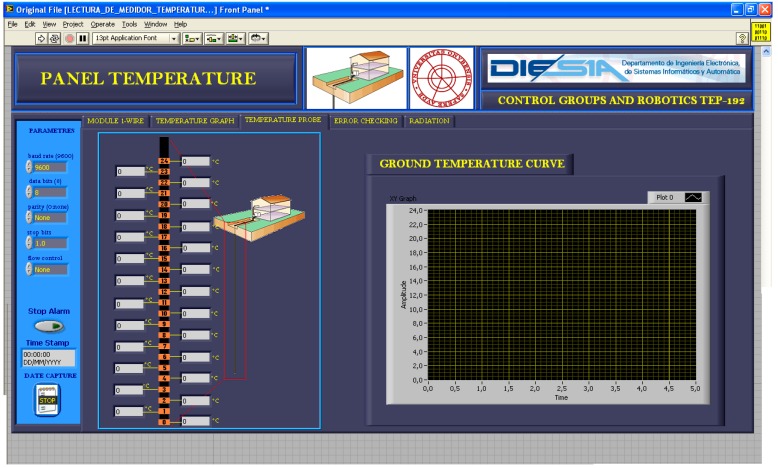
Real time temperature measurement of the 24 TS of the GTP.

**Figure 8:**
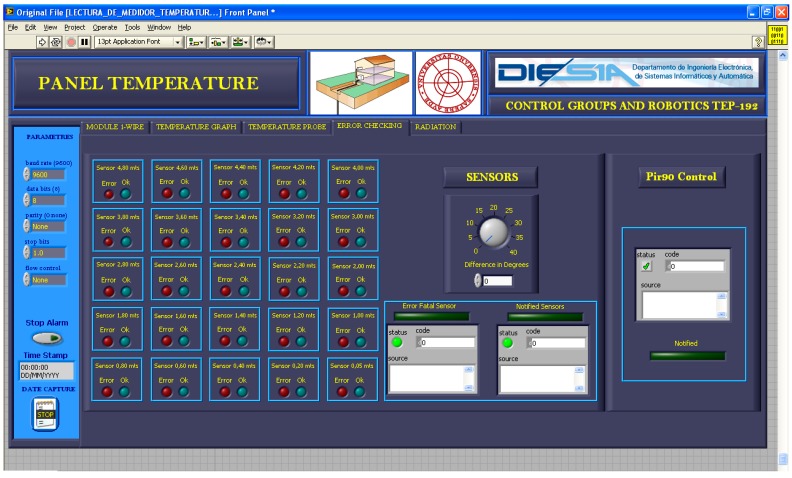
Screen that show faults in any TS of the GTP and allows adjustment of all parameters.

Figure 7 shows the VI screen with real-time measurement of the 24 TS of the GTP. Figure 8 shows the VI screen to detect the failure of a sensor. Failure detection is performed by comparison between adjacent sensors. The temperature gap is adjustable for each application and when it is exceeded an alarm is triggered. When a sensor malfunction it can be disconnected remotely from the VI thanks to developed electronics (see Figure 6).

## 4. Experimentation and Results

In order to put into practice and test the developed method and instrumentation system, we have had the opportunity to participate in the construction of a chalet with basement located at the southwest of Spain. Here we have taken soil temperature measurements with the developed GTP for about a year. At the building site local climate is warm tempered by the influence of the Atlantic Ocean with an annual average temperature around 19 °C. The coldest month is usually January (some years December or February), with an average temperature around 12 °C, and the warmest July (some years August), with an average temperature around 30 °C. The annual rainfall is around 500 mm and it has over 3000 h of sunshine and only about 60 cloudy days per year.

**Figure 9:**
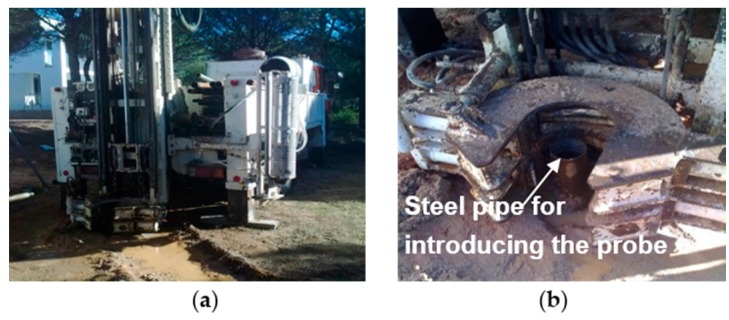
(**a**) Drilling machine; (**b**) Steel pipe for introducing the probe.

The chalet construction work has served as a real laboratory for about a year. In Figure 9a geotechnical drilling machine that was also exploited to make the drill through which the probe was introduced into a steel pipe (Figure 9b) is shown. Then, once the probe was placed in its final position, the steel pipe was removed. Later the holes between the probe and the ground were filled and compacted carefully. Finally the perimeter was watered abundantly, in order to favor the GTP contact with the ground along its whole length. The measurement campaign began when this artificial humidity was dissipated, several days later.

**Figure 10:**
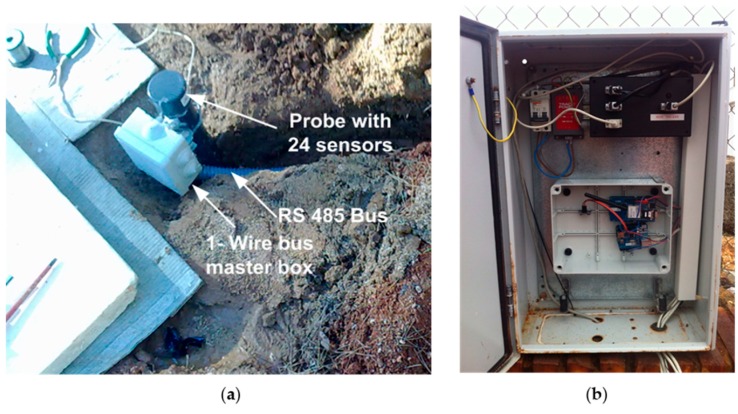
(**a**) Details of the buried GTP tip, box which houses the 1-wire bus master and RS 485 bus; (**b**) Waterproof box housing remainder elements of the measurement system.

The initial end of the probe, once buried, remains outside (see Figure 10a). The 1-wire bus that picks up signals from the 24 TS (see Figure 4 and Figure 5) goes from the GTP to a waterproof box (protection category IP 65) which houses the 1-wire bus master. From here the RS 485 bus goes to another larger size waterproof box which houses the remaining elements of Figure 4 (see Figure 10b).

**Figure 11:**
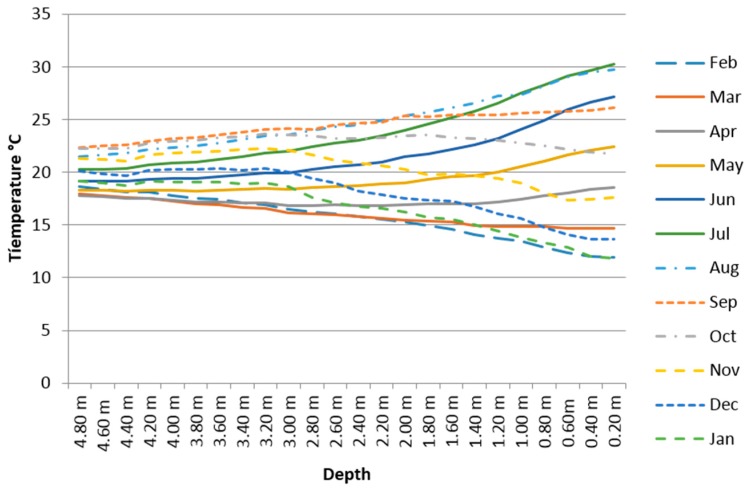
Behavior of the average monthly temperature regarding soil depth.

The buried GTP has been acquiring data for about a year, continuously (six measurements per minute) from 12 months (February 2103 to January 2014). Figure 11 shows the average monthly temperatures regarding depth measured by the GTP. Note clearly that as the depth increases the soil temperature tends to the annual average temperature of the place ($T_{m}$ in Equation (4) and 19.9 °C measured in the considered time period). In fact a temperature annual gap around 20 °C in the surface becomes only around 5 °C at 4.80 m depth.

**Figure 12:**
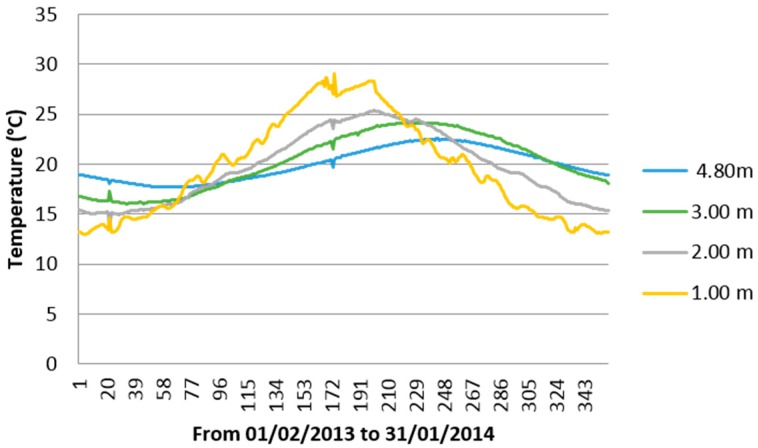
Average daily temperatures at different depths from 1 m to 4.8 m.

Figure 12 shows for the same time period the average daily temperatures at different depths (from 1 m to 4.8 m). Notice the sinusoidal profile as we have explained in Section 2 of this paper and how just 4.80 m deep, soil temperature is close all year to the annual average temperature in the place (19.9 °C). Note also the timing difference (phase shift) between sinusoidal functions which is due to the soil thermal inertia. This is also applicable to Figure 11 in the sense that the behavior of a surface average monthly temperature does not correspond with that expected for this month as the depth increases.

As we can observe in Figure 12 the sinusoidal profile regarding depth is each time better, which indicates that the influence of the surface weather decreases with depth, so the temperature profile is each time softer and closer to a sinusoid. This is also applicable to Figure 11.

### Calculation of the Actual Ground Thermal Diffusivity

In order to probe the power and usefulness of the developed methodology and system we have entrusted to a specialized company preparation of a geological report of the soil in the experimentation place regarding depth. This report asserts that from surface to a 4.80 depth there are five different kind of soil. In particular at 4.80 m depth the soil is a mix of sand and gravel. Moreover, the report asserts that the ground water level is 5.20 m depth so at 4.80 m the soil is wet.

Table 1 (our own elaboration from [10]) shows thermal conductivity, volumetric heat capacity and thermal diffusivity for different kinds of soil. With the help of Table 1 and the data obtained in the geological report we must decide the ground thermal diffusivity coefficient to use. However, depending on the amount of humidity and the mix of gravel and sand the range varies from $0.19\times 10^{-6}\;m^{2}/\text{s to }1.72\times 10^{-6}\;m^{2}/s$. It is clearly too high a range to decide without any further criteria.

At this stage it is where we see the power and usefulness of the developed methodology and system (even more if we had not geological report). Thus, from Equation (8), with $T_{m}$ (19.9 °C) and $T_{p}$ (12 °C) measured in the place and with *T_H_* (22.5 °C) measured by GTP, we can calculate the real ground thermal diffusivity value to 4.80 m depth. This is:

(9) $$\alpha_{z}=\frac{\pi }{T}{\left(\frac{z}{\text{ln }\frac{T_{p}}{T_{H}-T_{m}}}\right)}^{2}=\frac{\pi }{3.1557\;\times 10^{7}}{\left(\frac{4.8}{ln\;\frac{12}{22.5-19.9}}\right)}^{2}=0.98\;\times 10^{-6}\frac{m^{2}}{s}$$

The result corresponds, as we can expect, with a mixed sand and gravel soil and with a determined moisture grade. This composition and soil state is irrelevant to us (for this application), because what really matters is the value of $\alpha_{z}$ to 4.80 m depth $\left(\alpha_{4.80}\right)$.

## 5. Conclusions

This paper tries to reflect a couple of year of work of studying, analyzing and experimenting with soil temperature measures in order to find a practical and low cost way to dispose of the soil principal variable measurements (temperature and ground thermal diffusivity) to design VLEGE systems in a proper way. The studies conducted and conclusions reached are not only applicable in VLEGE systems, but also in construction, agriculture, biology, *etc.*

Focusing on VLEGE systems, the fluid/ground heat exchange lies in the fact that the temperature difference between the incoming fluid (e.g., air) and the ground varies during the year and depends on the depth to which the collector (pipe) is buried. The amplitude of the ground temperature variation decreases exponentially with depth and it tends to stabilize to the average temperature of the place, therefore at greater depth higher heating/cooling gain.

The methodology and instrumentation system developed in this work can be a useful tool for architects, geologists and engineers, since it gets the real values of the soil variables contrary to exclusively theoretical or empirical methodologies. Incorrect sizing of the VLEGE facility cause malfunction and poor performance of the heat pump. This is a serious problem: dig up collectors and change them is expensive and sometimes, depending on the characteristics of the facility, impossible. Consequently, this may result in the need to use external heating/cooling, which call into serious question the energy efficiency of the facility.

It seems that in an actual construction work is not always possible to perform a one year long ground temperature measurement campaign. This is true, however by observing Figure 3 and Figure 12 is easily verified that is not necessary. The sinusoidal behavior of the soil temperature implies that one needs only a part of the function to rebuild it fully. Really one only needs to get data around *T_H_* or *T_L_* and it is easy because you know (in basis on the timing difference or phase shift between the maximum/minimum soil temperature at chosen depth and on the surface) the days of the year when that will happen.

## Figures and Tables

**Table 1:** Thermal conductivity, volumetric heat capacity and thermal diffusivity for different kinds of soil.

Rock Type	Thermal Conductivity (W/mK)			Volumetric Heat Capacity (MJ/m^3^K)	Thermal Diffusivity (10^6^ m^2^/s)		
	Min	Typ	Max		Min	Typ	Max
Basalt	1.3	1.7	2.3	2.6	0.5	0.65	0.88
Greenstone	2	2.6	2.9	2.9	0.69	0.90	1
Gabbro	1.7	1.9	2.5	2.6	0.65	0.73	0.96
Granite	2.1	3.4	4.1	3	0.7	1.13	1.37
Peridotite	3.8	4	5.3	2.7	1.41	1.48	1.96
Gneiss	1.9	2.9	4	2.4	0.79	1.21	1.67
Marble	1.3	2.1	3.1	2	0.65	1.05	1.55
Mica schist	1.5	2	3.1	2.2	0.68	0.91	1.41
Shale sedimentary	1.5	2.1	2.1	2.5	0.6	0.84	0.84
Limestone	2.5	2.8	4	2.4	1.04	1.17	1.67
Loam	1.5	2.1	3.5	2.3	0.65	0.91	1.52
Quartzite	3.6	6	6.6	2.2	1.64	2.73	3
Salt	5.3	5.4	6.4	1.2	4.42	4.5	5.33
Sandstone	1.3	2.3	5.1	2.8	0.46	0.82	1.82
Siltstones and argillites	1.1	2.2	3.5	2.4	0.46	0.92	1.46
Dry gravel	0.4	0.4	0.5	1.6	0.25	0.25	0.31
Water saturated gravel	1.8	1.8	1.8	2.4	0.75	0.75	0.75
Dry sand	0.3	0.4	0.55	1.6	0.19	0.25	0.34
Water saturated sand	1.7	2.4	5	2.9	0.59	0.83	1.72
Dry clay/silt	0.4	0.5	1	1.6	0.25	0.31	0.62
Water saturated clay/silt	0.9	1.7	2.3	3.4	0.26	0.5	0.68
Peat	0.2	0.4	0.7	3.8	0.05	0.10	0.18
